# Understanding the Link Between Maternal Overnutrition, Cardio-Metabolic Dysfunction and Cognitive Aging

**DOI:** 10.3389/fnins.2021.645569

**Published:** 2021-02-26

**Authors:** Daria Peleg-Raibstein

**Affiliations:** Laboratory of Neurobehavioural Dynamics, Institute for Neuroscience, Department of Health Sciences and Technology, ETH Zürich, Zurich, Switzerland

**Keywords:** maternal, obesity, overnutrition, cardiovascular disease, offspring, animal models, cognition, aging

## Abstract

Obesity has long been identified as a global epidemic with major health implications such as diabetes and cardiovascular disease. Maternal overnutrition leads to significant health issues in industrial countries and is one of the risk factors for the development of obesity and related disorders in the progeny. The wide accessibility of junk food in recent years is one of the major causes of obesity, as it is low in nutrient content and usually high in salt, sugar, fat, and calories. An excess of nutrients during fetal life not only has immediate effects on the fetus, including increased growth and fat deposition *in utero*, but also has long-term health consequences. Based on human studies, it is difficult to discern between genetic and environmental contributions to the risk of disease in future generations. Consequently, animal models are essential for studying the impact of maternal overnutrition on the developing offspring. Recently, animal models provided some insight into the physiological mechanisms that underlie developmental programming. Most of the studies employed thus far have focused only on obesity and metabolic dysfunctions in the offspring. These studies have advanced our understanding of how maternal overnutrition in the form of high-fat diet exposure can lead to an increased risk of obesity in the offspring, but many questions remain open. How maternal overnutrition may increase the risk of developing brain pathology such as cognitive disabilities in the offspring and increase the risk to develop metabolic disorders later in life? Further, does maternal overnutrition exacerbate cognitive- and cardio-metabolic aging in the offspring?

## Introduction

The prevalence of obesity is increasing worldwide and has reached epidemic proportions posing a major problem for healthcare (Ng et al., 2014; Hruby and Hu, 2015). The last World Health Organization (WHO) report classified 1.9 billion adults worldwide as overweight and more than 650 million as obese (WHO, 2020). Obesity is associated with an increased incidence of comorbidities like dyslipidemia, hypertension and type 2 diabetes mellitus (T2DM; Hruby and Hu, 2015). These conditions are associated with higher cardiovascular disease risk and mortality. A particular concern emerged in the past two decades, namely obesity during pregnancy (Heslehurst et al., 2010; Huda et al., 2010; Gregor et al., 2016; Lindberg et al., 2016; Kominiarek and Peaceman, 2017). Nearly two thirds of women of childbearing age (19–44) are overweight and 36.5% are obese. It is therefore not surprising that this epidemic also affects children of all ages with nearly 38 million children under the age of 5 years classified as overweight or obese (WHO, 2020). It is already known that the obesity epidemic cannot be explained only as a result of an affluent lifestyle, reduced physical activity or a genetic predisposition (Albuquerque et al., 2017; Sheikh et al., 2017). Evidence has suggested that it may originate from environmental factors present already early in development during fetal life. In the early 1980s the Barker hypothesis, also referred as the “developmental origins of adult disease,” alluded to an association between fetal undernutrition and low birth weight with the risk of developing adult obesity, including the metabolic syndrome and cardiovascular disease (Barker et al., 1990; Barker, 2007). Programming is a process whereby an insult at a critical time period of development has lifelong significance. According to the Barker hypothesis, variations in the transfer of food from the mother to her baby have profound and long-term implications for the health of the next generation (for review see McMillen et al., 2005). Since the original observations of David Barker, various human and animal studies shifted their investigations to studying the effects of maternal overnutrition and obesity, since diets high in calories and fats combined with limited physical activity and increased sedentary behavior have become more prevalent in developed as well as under-developed countries. Conforming to this hypothesis, studies in the past two decades suggested a link between maternal obesity and/or overnutrition during pregnancy and the increase risk of obesity later in life (Kominiarek and Chauhan, 2016). Epidemiological studies report a positive relationship between weight at birth and adult body mass index (BMI). Moreover, maternal BMI, in particular its increase during pregnancy is positively related to obesity in the offspring as babies, through childhood and into adulthood (Lawlor et al., 2007). More specifically, in a United States cohort, maternal first-trimester obesity led to a two- to threefold increase in the risk of childhood obesity in their progeny; 24% of the children of obese mothers were themselves obese at age 4, compared with 9% of the children of normal-weight mothers (Whitaker, 2004). A maternal hypernutritional state, due to pre-existing T2DM, gestational diabetes or obesity, generates a long-term risk of obesity for the child. Gestational weight gain, irrespective of pre-pregnancy body mass, is positively associated with obesity in 3 years old children (Oken et al., 2007). Maternal obesity increases a child’s risk for developing obesity as a consequence of either shared genes, environmental factors, or a combination of both. Parents not only create food environments for their children but also influence their eating behaviors, taste preferences, and food choices (Kral and Rauh, 2010). The *in utero* environment, which profoundly affects the fetal developmental processes, is considered as important as genes or family habits in determining the predisposition to long-term health outcomes. Thus, the *in utero* environment is a key player leading to increased risk of obesity, T2DM and cardiovascular disease in the adult offspring upon exposure to increased nutrient supply before birth (Reynolds et al., 2013; Godfrey et al., 2017). A relation between different insults during pregnancy, including famine, different types of infections, prenatal stress, obstetric complications, smoking, were shown to impact the adult life of the progeny (Boksa, 2004). However, it appears virtually impossible in human studies to establish a cause-effect relationship, to disentangle the direct effects of maternal obesity from the influence of shared genes and postnatal lifestyle on the developing child. Therefore, animal models are necessary to answer these crucial questions and generate results that then may be translated to humans.

This review will summarize findings on the long-term effects of maternal overnutrition and obesity on metabolic states and cognitive function in the offspring from human and rodent studies. It will also try to identify whether one trait can be the consequence of the other.

## The Maternal Overnutrition/Obesity Animal Model

Maternal obesity can be induced by exposing female animals to different nutritional diets for example hyperenergetic and highly palatable diets such as high-fat (varying between 45 and 60% calories from fat) (Tozuka et al., 2009, 2010; Peleg-Raibstein et al., 2012, 2016; Kang et al., 2014; Graf et al., 2016; Janthakhin et al., 2017; Robb et al., 2017; Sarker and Peleg-Raibstein, 2018; Sarker et al., 2018, 2019a,b; Wolfrum and Peleg-Raibstein, 2018; Moreton et al., 2019; Zieba et al., 2019), a combination of high-fat high-sugar diet, high-sugar diet or a cafeteria diet which supplement the normal chow diet with a variety of palatable foods (Wright et al., 2014; Ribeiro et al., 2018; Lewis et al., 2019; Moreton et al., 2019). These different diets promote weight gain and depending on the length of exposure induce obesity and other metabolic disorders to the mother. These diets are trying to mimic the human modern food habits and consumption. Each of these types of diets have their advantages and disadvantages. For example, in the high-fat diet (HFD) and high-fat high-sugar diet (HFHS) models, the macronutrients and micronutrients contents can be controlled between the obesogenic diets and the control chow diet. They are commercially available and can be easily utilized. Whereas, the cafeteria diet model has different variations depending on the laboratory and can be tailored depending on the specific research question and adapted accordingly. Female mice exposed to the cafeteria diet gain faster body weight and develop increased body fat composition with changes of other metabolic markers (i.e., insulin levels, triglycerides levels etc.) compared to the HFD model. However, in this diet it is more difficult to control for intake of macronutrients and micronutrients. In addition, by using this diet one needs more careful planning and executing of the diet schedules because otherwise the changes in diet composition throughout the experiment will have too much variations.

An overview of the current literature on animal studies investigating the effects of maternal obesity or overnutrition utilizes different maternal diets (as mentioned above) as well as different exposure time periods such as exposure only during gestation, only during lactation or prior to mating (conception) and during gestation and lactation (Ghosh et al., 2001; Siemelink et al., 2002; Buckley et al., 2005; Gregersen et al., 2005; Khan et al., 2005; Chen et al., 2008, 2009; Naef et al., 2008, 2011; Dunn and Bale, 2009, 2011; Elahi et al., 2009; Morris and Chen, 2009; Niculescu and Lupu, 2009; Tamashiro et al., 2009; White et al., 2009; Bilbo and Tsang, 2010; Chechi et al., 2010; Gregorio et al., 2010; Vucetic et al., 2010; Ashino et al., 2011; Dunn et al., 2011; Simar et al., 2011; Strakovsky et al., 2011; Wahlig et al., 2011; Zhang et al., 2011; Peleg-Raibstein et al., 2012, 2016; Vogt et al., 2014; Sarker and Peleg-Raibstein, 2018; Sarker et al., 2018, 2019a,b; Wolfrum and Peleg-Raibstein, 2018; Zieba et al., 2019). In addition, also the time periods of exposure prior to mating differ between the studies. Thus, these studies have led to considerably inconsistent metabolic and behavioral outcomes in the offspring.

## The Long Term-Effects of Maternal Overnutrition on Cognitive Function of the Offspring

With rates of global obesity constantly increasing, maternal obesity, and excessive gestational body weight leading not only to obesity but also to other long-term health outcomes such as cardiovascular disease and mental disorders including cognitive decline in the progeny. In the past few years, human studies linked maternal obesity with cognitive abnormalities in the offspring (for review, Van Lieshout, 2013; Contu and Hawkes, 2017). More specifically, maternal obesity was linked to reduction in child intelligence quotient (IQ) scores (Neggers et al., 2003; Gage et al., 2013; Bliddal et al., 2014; Pugh et al., 2015), cognitive test scores (Tanda et al., 2013), impaired neuropsychological development (Hinkle et al., 2012; Casas et al., 2013; Huang et al., 2014) and autism spectrum disorder and other intellectual disabilities (Brion et al., 2011; Li et al., 2016; Kong et al., 2020) in the children. Additionally, other studies suggested that an association might exist between maternal obesity and increased symptoms of attention-deficit hyperactivity disorder (ADHD) in children (Rodriguez et al., 2008; Rodriguez, 2010; Buss et al., 2012; Jenabi et al., 2019; Kong et al., 2020; Li et al., 2020; Robinson et al., 2020). Although the association between maternal obesity and offspring’s cognitive disabilities was described, the current literature is still scarce, and findings are inconsistent (Brion et al., 2011; Keim and Pruitt, 2012; Van Lieshout, 2013; Bliddal et al., 2014). The underlying mechanisms leading to a higher susceptibility of the progeny to develop cognitive abnormalities later in life is still unknown. The observed cognitive impairments in the adult offspring could also be a result of obesity. Therefore, the understanding how maternal weight and weight gain might contribute to offspring’s cognitive development is important, however, knowledge gaps remain. It was shown that excessive food intake and obesity leads to increased risk for cognitive impairments and to various types of neurodegenerative dementia (Stranahan and Mattson, 2008; Sellbom and Gunstad, 2012; Wraw et al., 2018). Therefore, to date, it is still not clear whether the cognitive decline is a trait that precede obesity and/or the metabolic syndrome, or whether the metabolic state itself is leading to cognitive disabilities. Another important question is whether maternal overnutrition exacerbate aging, metabolically and cognitively in subsequent generations.

Maternal nutrition is important for an optimal neurodevelopment of the offspring. In recent years, an increased interest emerged looking at the effects of maternal nutrition and offspring cognitive function. When assessing learning and memory, most of the studies investigated the effects of maternal overnutrition and obesity by examining hippocampal-dependent learning and memory mainly employing the Morris water maze paradigm. Bilbo and Tsang (2010) demonstrated that maternal diet high in either saturated or trans fats induced increased spatial memory performance and a concomitant increased inflammation within the hippocampus compared to control offspring. In contrast, decreased spatial memory performance in the Barnes maze was found in young and adult offspring born to obese mothers (Tozuka et al., 2010). Similar impaired spatial memory performances were observed in offspring exposed to a HFD throughout both the pre- and postnatal period (White et al., 2009; Page et al., 2014; Lépinay et al., 2015). Working memory assessment employing the novel object recognition task also lead to contradictory findings between studies. One study described reduced novel object exploration in young adult offspring exposed to maternal HFD (MHFD) (Graf et al., 2016) while offspring exposed to maternal cafeteria diet during lactation reported increased novel object exploration (Wright et al., 2014). Another study employing exposure led to a wide spectrum of cognitive abnormalities in an age-depend manner in the offspring (Wolfrum and Peleg-Raibstein, 2018). The behavioral abnormalities observed in the adult offspring readily suggests that the perturbations caused by MHFD exposure are diverse and fundamental to normal brain development. It was shown that offspring exposed to MHFD were severely impaired in acquisition of avoidance learning in an aversive learning task, a two-way active avoidance paradigm, as compared to control offspring at adulthood (Wolfrum and Peleg-Raibstein, 2018) while working memory and fear memory remained intact (Peleg-Raibstein et al., 2012; Wolfrum and Peleg-Raibstein, 2018) during adulthood and impaired in aged-adult HFD offspring (Wolfrum and Peleg-Raibstein, 2018). This study had several unique strengths: the longitudinal nature allowed to assess offspring cognition at different developmental ages utilizing different behavioral cognitive testing. The authors could pinpoint when different cognitive dysfunctions such as working memory, spatial memory and associative memory were evident. In summary all these findings can show that cognitive abilities can be influenced by obesity of the offspring. Most of these behavioral tests are dependent of hippocampus function (Vorhees and Williams, 2014). A brain region important in cognitive processing, learning and memory and that was also shown to be sensitive to changes in dietary energy intake (Miller and Spencer, 2014) and in aged-related cognitive ability (Gerstein et al., 2013). In addition, it was shown that exposure to a high-calorie diet in middle-aged rats led to impaired hippocampus-dependent cognitive functions such as spatial learning and which was accompanied by reduced hippocampal neurogenesis and synaptic plasticity (Stranahan et al., 2008). Since the development of the hippocampus is sensitive to *in utero* nutritional insults, it is not surprising that maternal HFD induced cognitive function impairments which was associated with inhibition of hippocampal neurogenesis and increased apoptosis in the offspring (Tozuka et al., 2009; Kim and Park, 2018).

These discrepancies might be due to methodological differences between the studies such as different gender used, timing of maternal dietary exposure, strain of the animals, different maternal diets employed, different testing protocols and age of the offspring at the time of testing. Another important consideration for the interpretation of the findings is that not all of the maternal diets utilized induce differences in gestational weight between female dams exposed to diet-induced obesity or control diet with no difference in any other metabolic parameters [i.e., fat mass, plasma glucose, insulin, triglycerides, cholesterol, and FFA levels (Peleg-Raibstein et al., 2016)]. While other animal models of maternal obesity lead to an obese state of the mothers (Bilbo and Tsang, 2010; Tozuka et al., 2010; Simar et al., 2011). This makes it difficult to dissociate between the direct effect of maternal obesity and that of overnutrition leading to obesity in the offspring.

Until now, relatively little is known about how maternal overnutrition/obesity can lead to lifelong consequences in cognitive abilities of the offspring. In this respect, this field of research is still in its early stages, and additional studies are required to examine the long-term effects of maternal overnutrition/obesity on memory and learning abilities of the offspring that may lead to advanced cognitive aging and also to increased risk to develop Alzheimer’s disease (AD).

## Nutritional Programming of Alzheimer’s Disease and Related Cognitive Decline

To this date, investigations on the impact of maternal overnutrition and maternal obesity on offspring cognitive performance and mental health in animal models have focused mainly on anxiety, depression and motivation with some newer studies also examining the effects on learning and memory. Alzheimer’s disease, the most common form of dementia, is a chronic progressive neurodegenerative disorder that develops slowly over decades. The observable pathological brain changes and the symptoms often do not follow the same time-course, which makes the diagnosis difficult. One of the clinically observed symptoms is memory loss, with the hippocampus and the cerebral cortex being the brain regions primarily involved. Interestingly, classic clinical studies indicate that human AD is also associated with damage in brain areas related to nutrient-sensing and the motivation to move, especially the hypothalamus which is also linked to the ability to form memories (Ishii, 1966; Saper and German, 1987). The exact etiology of AD is still not fully understood, however, ample evidence points to amyloid-beta peptide (Aβ) as a key player in the pathogenesis of AD and is the earliest lesion in the disease process (Hardy and Higgins, 1992; Ballard et al., 2011). In the past, AD was thought to be a disease that developed in later life, but there is increasing evidence that the disease probably begins many years before clinical symptoms appear. Thus far, very little is known about this “silent” stage of the disease. Exactly when AD begins, and why some people get it and others do not, is still not fully understood. There are many environmental risk factors, such as nutrition, that are thought to have their major effects long before the disease can be diagnosed (Killin et al., 2016). For example, elevated dietary intake of fat has been shown to increase the risk of developing AD and facilitate age-related cognitive impairments (Luchsinger et al., 2002; Hooijmans and Kiliaan, 2008; Gustafson, 2012). Therefore, it is not surprising that obesity and consumption of a Western-style diet, especially in mid-life, are associated with increased risk of AD later in life (Laitinen et al., 2006). The prevalence of AD is greater in countries with a high intake of high-fat/high-calorie diets and lower in those that consume low-fat diets (Grant, 1999; Panza et al., 2004). Recently, evidence suggests that maternal diet may also impact accelerating aging and (McAninch et al., 2020) the subsequent appearance of AD in late life (Borenstein et al., 2006; Lahiri et al., 2008; Miller and O’Callaghan, 2008; Tolppanen et al., 2016). In the previous section we discussed the association from human studies and evidence from preclinical animal models between maternal obesity and unhealthy eating with cognitive impairments and disturbances of cerebral cortex and hippocampus function (Van Lieshout et al., 2011; Van Lieshout, 2013; Cordner and Tamashiro, 2015; Contu and Hawkes, 2017). These leads to neuropsychological impairments as deficits in attention, working memory and executive function. However, to date the underlying mechanisms linking maternal diet to these pathophysiological changes of the brain and behavior are not fully understood. As obesity is a risk factor for AD and excessive intake of fats in adulthood worsen AD in animal models (Tolppanen et al., 2016; Baranowski et al., 2018; Lloret et al., 2019), it is possible that maternal overnutrition affects the development of AD in the offspring. Animal models are fundamental for our understanding the effects of an obesogenic environment during fetal life and the effects depend on multiple and not-exclusive pathways that may lead to long-term neuropathological outcomes. Thus, by employing the overnutrition/obesity animal model with a common genetic background, carefully controlled dietary and activity conditions and a controlled postnatal environment is fundamental for examining how overnutrition prior and during pregnancy increases the development of obesity, cardiometabolic disease and the risk to develop AD in the offspring. Thus far only a handful of preclinical studies investigated the impact of maternal overnutrition/obesity on AD-like cognitive pathology. It was shown that MHFD led to impairment in memory in 2- and 12-month-old triple transgenic mouse model of AD (3xTgAD) offspring compared to control offspring (Martin et al., 2014). The memory impairments were accompanied by a significant increase in the number of hippocampal tau positive neurons. These findings may imply that MHFD can induce the onset and progression of AD later in life (Martin et al., 2014). In addition, it was demonstrated that the pathological AD marker, clearance of the β-amyloid peptide, was impaired in brains of MHFD offspring (Hawkes et al., 2015). Additionally, offspring born to MHFD Tg2576 mothers (i.e., the Tg2576 mouse model of AD which express the Swedish mutation in the human amyloid precursor protein) developed higher levels of hippocampal β-amyloid pathology compared to control offspring (Nizari et al., 2016).

## A Link Between Central Lipids and Cognitive Function?

The brain is the most cholesterol rich organ in the body. The majority of central cholesterol accumulates during embryonic development and in the early postnatal period, while the metabolism of cholesterol in adulthood is characterized by a low turnover and minimal loss of cholesterol (Zhang and Liu, 2015). Brain cholesterol is proposed to be derived by *de novo* synthesis, since only small amounts of plasma cholesterol can transfer through the blood–brain barrier (BBB; Dietschy and Turley, 2001). Cholesterol supply to the central nervous system (CNS) is believed to be mediated by astrocytes and microglia, which predominantly secrete cholesterol and phospholipids together with apolipoprotein E (ApoE). ApoE, a protein involved in fats metabolism in the body, in turn serves as a ligand for these lipoproteins to affect the uptake of lipoproteins via the low-density lipoprotein receptor (LDLR) and the lipoprotein related protein (LRP; Lane-Donovan et al., 2014). Currently it is believed that ApoE-containing lipoproteins redistribute lipids and regulate cholesterol homeostasis within the brain (Mahley, 2016b). As cholesterol is essential for normal brain function including learning and memory, dysfunction in central cholesterol metabolism might lead to deficiencies that will induce structural and functional brain disorders such as AD (Kim et al., 2009a; Mahley, 2016a; Yamazaki et al., 2019). In this context, the expression of ApoE in the brain concomitant with LDLR function has been implicated. For example, one study demonstrated that overexpression of ApoE in the brain had beneficial effects on cognitive function as well as neural circuit function by enhancing the clearance of Aβ (Cramer et al., 2012). Furthermore, LRP1 forebrain knockout mice show alterations in central lipid metabolism in brain regions important for cognitive function paralleled with impairments in memory (Liu et al., 2010). In addition, neuron-specific 2 (LPL) deficient mice display learning and memory deficits (Xian et al., 2009) and overexpression of brain LDL receptors reduces amyloid deposition and may represent a novel treatment for AD (Kim et al., 2009b). Thus, current evidence points to the fact that central cholesterol homeostasis is important for cognitive function. However, the link to peripheral cholesterol metabolism is not yet understood. More specifically, it remains unknown, by which mechanisms lipoproteins are produced in the brain and whether there may be a transport or a link to cholesterol precursors across the BBB thus connecting central and systemic lipid homeostasis?

## The Effects of Maternal HFD Exposure and the Increased Risk for Cardio-Metabolic Disease in the Offspring

In human epidemiological studies maternal BMI was positively correlated with cardiovascular events and premature death in the progeny (Reynolds et al., 2013). Maternal overnutrion/obesity was shown to induce a cardiometabolic phenotype (increased fat disposition, alterations in plasma metabolic markers, increased body weight, hyperinsulinemia, and fasting glucose levels) (Godfrey et al., 2017). In preclinical studies offspring born to over nourished mothers showed increased risk to develop cardiovascular pathology compared to their control counterparts (Samuelsson et al., 2008; Liang et al., 2009; Blackmore et al., 2014; Loche et al., 2018).

In order to try to investigate how maternal overnutrition/obesity influences the cardio-metabolic function of the offspring the body weight is not always a predictor of body composition. Some studies have shown that, despite similar body weights of the offspring of over nourished mothers and control offspring, elevated percentages of body fat can be detected (Buckley et al., 2005; Howie et al., 2009). These findings suggest that elevated body fat percentages may be more likely a result of reduced lean mass than an increase in fat mass *per se*. The pathophysiological processes underlying a cardio-metabolic state (obesity, diabetes, and cardiovascular dysfunction), cognitive decline and structural brain changes are most likely multifactorial. There are some evidence that inflammation (i) may be the cause of decline in cognitive processes in aging (Cornejo and von Bernhardi, 2016), (ii) is a risk factor for acceleration in cognitive decline (Stacey et al., 2015), and (iii) may be a cause for neurodegenerative disorders (Schain and Kreisl, 2017). Inflammation may cause alterations in brain structures such as reduced hippocampal and prefrontal volume (Bruehl et al., 2009), decrease in white matter (Kullmann et al., 2016), and reduction in total brain volume (Jefferson et al., 2007). In addition, inflammation processes were also associated with obesity and the metabolic syndrome (Guillemot-Legris and Muccioli, 2017). Taken together, these findings may suggest that one potential mechanism for explaining the exacerbation of cognitive decline observed in the offspring is alteration in pro-inflammatory markers in plasma and/or brain (Contu and Hawkes, 2017). Expression of brain pro-inflammatory markers such as TNFα, IL1, and IL-6 will in brain structures that predominantly underline memory, learning, and attentional processes, such as the hippocampus, prefrontal cortex, and the hypothalamus in different age stages of the offspring will enable the identifications of how longitudinal changes in inflammation markers might correlate with cognitive acceleration and/or cardiovascular dysfunction. C-reactive protein (CRP) is acting as an indicator for inflammation. In humans increased levels of circulation CRP was associated with reduced cognitive function and is used as a marker to predict future risk for stroke and ischemic attack (Kuo et al., 2005).

There is still very little known on the role of circulating lipoproteins and cholesterol on neuropathological disorders, such as aging and cognitive decline on the one hand and increased risk to develop cardiovascular disorders on the other hand. Enhancing our understanding on the role and function of lipoproteins and cholesterol in the brain and their transport to the brain, may have an enormous impact in this field. In addition, a potential target for treatment for age-related cognitive and cardio-metabolic disabilities.

## Conclusions and Future Directions

The epidemic of obesity is spreading fast through developed and developing countries. The latest projections from the WHO predict that 1 in 10 is obese (this is an average as in developed countries it is considerably higher). Obesity is a dangerous condition because it creates a permissive environment for many other health-related diseases, such as type 2 diabetes, hypertension and heart disease. Even more worrying are the facts that health problems not only affect the adult population, but also children (Ogden et al., 2014). Childhood obesity is of major concern because it will most likely be translated to higher rates of adult obesity and related comorbidities such as diabetes and cardiovascular disease (Freedman et al., 2007; Li et al., 2008; Pulgarón, 2013). Growing evidence in the past two decades has shown that maternal overnutrition and/or obesity has a long-term impact on offspring health, demonstrating that the *in utero* environment might be a key determinant of long-term health outcomes. However, that far epidemiological human studies cannot distinguish between the effect of overnutrition during pregnancy and postnatal nutrition (such as an obesogenic household). Many human studies investigating the long-term implications of developmental insults on the health of the offspring (such as famine or infection), can point to causality since the insults occur during a specific point in time, while the rest of the pregnancy and childhood is normal. In the case of maternal overnutrition in humans, there is chronic consumption of palatable food during pregnancy that continues into childhood and adolescence, thereby preventing a conclusive association between the metabolic phenotype of the adult offspring exposed to prenatal overnutrition. There are several other limitations to human observational studies due to methodological differences in the publish research that make conclusion difficult or impossible such as socio-economic factors, adjustment for maternal intelligence, inadequate reporting of nutrition evaluations (through self-reported questionnaires), sample selection, low power due to a small sample size, etc. Thus, the establishment of the effect of maternal overnutrition on the offspring can only be studied in isolation in animal models. The health consequences of *in utero* exposure to maternal overnutrition on future generations are thus an area of intense research.

Until now, there are a few intervention studies utilizing the maternal obesity animal model. Those studies employ dietary (for review Zambrano et al., 2010; Nathanielsz et al., 2013; Kang et al., 2014; Liu et al., 2020), physical exercise (Carter et al., 2012; Vega et al., 2015; Fernandez-Twinn et al., 2017)[151; 152; 153], or therapeutic/supplementation interventions during different developmental stages (Vickers et al., 2005; Sen and Simmons, 2010). Some of these studies report potential beneficial effects on metabolic and cognitive outcomes in the offspring (Vickers et al., 2005; Vickers and Sloboda, 2012). Different interventions, a combination of approaches and the time of intervention may act through different mechanisms, which limit the intervention strategies to prevent the detrimental effects of maternal obesity. To date there are only a handful of interventional human studies that try to mitigate the health adversities in the offspring due to maternal obesity (Han et al., 2012; Briley et al., 2014; Chiswick et al., 2015; Poston et al., 2015, 2017; Thangaratinam, 2015; Dodd et al., 2016).

Prevention of obesity in women of childbearing age and the prevention of obesity during childhood are essential to fight the global obesity epidemic. Further, it is of outmost importance to elucidate the specific mechanisms linking *in utero* exposure to an hyperphagic diet to the development of obesity as well as cardiovascular disorders. The latter is the number one cause of global mortality. Outcomes from animal studies have a potential to unravel novel and yet unknown mechanisms involved in the pathophysiology of cardio-metabolic disease and how they are linked to cognitive impairments. A deeper understanding of whether and how maternal overnutrition exerts noxious effects on the health of the offspring may allow the identification of new targets for future interventions against the development of obesity-related cardiovascular disorders and neuropathology.

## Author Contributions

DP-R conceptualized the project, conducted the systemic review of the literature, and wrote the manuscript.

## Conflict of Interest

The author declares that the research was conducted in the absence of any commercial or financial relationships that could be construed as a potential conflict of interest.

## References

[B1] Albuquerque D. Manco L. Nóbrega C. Padez C. (2017). The contribution of genetics and environment to obesity. *Br. Med. Bull.* 123 159–173. 10.1093/bmb/ldx022 28910990

[B2] Ashino N. G. Saito K. N. Souza F. D. Nakutz F. S. Roman E. A. Velloso L. A. (2011). Maternal high-fat feeding through pregnancy and lactation predisposes mouse offspring to molecular insulin resistance and fatty liver. *J. Nutr. Biochem.* 23 341–348. 10.1016/j.jnutbio.2010.12.011 21543214

[B3] Ballard C. Gauthier S. Corbett A. Brayne C. Aarsland D. Jones E. (2011). Alzheimer’s disease. *Lancet (Lond. Engl.)* 377 1019–1031. 10.1016/S0140-6736(10)61349-9 21371747

[B4] Baranowski B. J. Bott K. N. MacPherson R. E. K. (2018). Evaluation of neuropathological effects of a high-fat high-sucrose diet in middle-aged male C57BL6/J mice. *Physiol. Rep.* 6:e13729. 10.14814/phy2.13729 29890051 PMC5995310

[B5] Barker D. J. (2007). The origins of the developmental origins theory. *J. Intern. Med.* 261 412–417. 10.1111/j.1365-2796.2007.01809.x 17444880

[B6] Barker D. J. Bull A. R. Osmond C. Simmonds S. J. (1990). Fetal and placental size and risk of hypertension in adult life. *BMJ (Clin. Res. ed.)* 301 259–262. 10.1136/bmj.301.6746.259 2390618 PMC1663477

[B7] Bilbo S. D. Tsang V. (2010). Enduring consequences of maternal obesity for brain inflammation and behavior of offspring. *FASEB J.* 24 2104–2115. 10.1096/fj.09-144014 20124437

[B8] Blackmore H. L. Niu Y. Fernandez-Twinn D. S. Tarry-Adkins J. L. Giussani D. A. Ozanne S. E. (2014). Maternal diet-induced obesity programs cardiovascular dysfunction in adult male mouse offspring independent of current body weight. *Endocrinology* 155 3970–3980. 10.1210/en.2014-1383 25051449 PMC4255219

[B9] Bliddal M. Olsen J. Støvring H. Eriksen H. L. Kesmodel U. S. Sørensen T. I. (2014). Maternal pre-pregnancy BMI and intelligence quotient (IQ) in 5-year-old children: a cohort based study. *PLoS One* 9:e94498. 10.1371/journal.pone.0094498 24727836 PMC3984139

[B10] Boksa P. (2004). Animal models of obstetric complications in relation to schizophrenia. *Brain Res. Brain Res. Rev.* 45 1–17. 10.1016/j.brainresrev.2004.01.001 15063096

[B11] Borenstein A. R. Copenhaver C. I. Mortimer J. A. (2006). Early-life risk factors for Alzheimer disease. *Alzheimer Dis. Assoc. Disord.* 20 63–72. 10.1097/01.wad.0000201854.62116.d7 16493239

[B12] Briley A. L. Barr S. Badger S. Bell R. Croker H. Godfrey K. M. (2014). A complex intervention to improve pregnancy outcome in obese women; the UPBEAT randomised controlled trial. *BMC Pregnancy Childbirth* 14:74. 10.1186/1471-2393-14-74 24533897 PMC3938821

[B13] Brion M. J. Zeegers M. Jaddoe V. Verhulst F. Tiemeier H. Lawlor D. A. (2011). Intrauterine effects of maternal prepregnancy overweight on child cognition and behavior in 2 cohorts. *Pediatrics* 127 e202–e211. 21187310 10.1542/peds.2010-0651 PMC3605781

[B14] Bruehl H. Wolf O. T. Sweat V. Tirsi A. Richardson S. Convit A. (2009). Modifiers of cognitive function and brain structure in middle-aged and elderly individuals with type 2 diabetes mellitus. *Brain Res.* 1280 186–194. 10.1016/j.brainres.2009.05.032 19463794 PMC2749480

[B15] Buckley A. J. Keseru B. Briody J. Thompson M. Ozanne S. E. Thompson C. H. (2005). Altered body composition and metabolism in the male offspring of high fat-fed rats. *Metabolism* 54 500–507. 10.1016/j.metabol.2004.11.003 15798958

[B16] Buss C. Entringer S. Davis E. P. Hobel C. J. Swanson J. M. Wadhwa P. D. (2012). Impaired executive function mediates the association between maternal pre-pregnancy body mass index and child ADHD symptoms. *PLoS One* 7:e37758. 10.1371/journal.pone.0037758 22719848 PMC3376097

[B17] Carter L. G. Lewis K. N. Wilkerson D. C. Tobia C. M. Ngo Tenlep S. Y. Shridas P. (2012). Perinatal exercise improves glucose homeostasis in adult offspring. *Am. J. Physiol. Endocrinol. Metab.* 303 E1061–E1068. 22932781 10.1152/ajpendo.00213.2012 PMC3469606

[B18] Casas M. Chatzi L. Carsin A. E. Amiano P. Guxens M. Kogevinas M. (2013). Maternal pre-pregnancy overweight and obesity, and child neuropsychological development: two Southern European birth cohort studies. *Int. J. Epidemiol.* 42 506–517. 10.1093/ije/dyt002 23569191

[B19] Chechi K. Herzberg G. R. Cheema S. K. (2010). Maternal dietary fat intake during gestation and lactation alters tissue fatty acid composition in the adult offspring of C57Bl/6 mice. *Prostaglandins Leukot. Essent. Fatty Acids* 83 97–104. 10.1016/j.plefa.2010.06.001 20688254

[B20] Chen H. Simar D. Lambert K. Mercier J. Morris M. J. (2008). Maternal and postnatal overnutrition differentially impact appetite regulators and fuel metabolism. *Endocrinology* 149 5348–5356. 10.1210/en.2008-0582 18635655

[B21] Chen H. Simar D. Morris M. J. (2009). Hypothalamic neuroendocrine circuitry is programmed by maternal obesity: interaction with postnatal nutritional environment. *PLoS One* 4:e6259. 10.1371/journal.pone.0006259 19606226 PMC2707610

[B22] Chiswick C. Reynolds R. M. Denison F. Drake A. J. Forbes S. Newby D. E. (2015). Effect of metformin on maternal and fetal outcomes in obese pregnant women (EMPOWaR): a randomised, double-blind, placebo-controlled trial. *Lancet Diabetes Endocrinol.* 3 778–786. 10.1016/s2213-8587(15)00219-3 26165398 PMC4673088

[B23] Contu L. Hawkes C. A. (2017). A review of the impact of maternal obesity on the cognitive function and mental health of the offspring. *Int. J. Mol. Sci.* 18:1093. 10.3390/ijms18051093 28534818 PMC5455002

[B24] Cordner Z. A. Tamashiro K. L. (2015). Effects of high-fat diet exposure on learning & memory. *Physiol. Behav.* 152 363–371. 10.1016/j.physbeh.2015.06.008 26066731 PMC5729745

[B25] Cornejo F. von Bernhardi R. (2016). Age-Dependent changes in the activation and regulation of microglia. *Adv. Exp. Med. Biol.* 949 205–226. 10.1007/978-3-319-40764-7_10 27714691

[B26] Cramer P. E. Cirrito J. R. Wesson D. W. Lee C. Y. Karlo J. C. Zinn A. E. (2012). ApoE-directed therapeutics rapidly clear beta-amyloid and reverse deficits in AD mouse models. *Science (New York, N.Y.)* 335 1503–1506. 10.1126/science.1217697 PMC3651582 22323736

[B27] Dietschy J. M. Turley S. D. (2001). Cholesterol metabolism in the brain. *Curr. Opin. Lipidol.* 12 105–112. 11264981 10.1097/00041433-200104000-00003

[B28] Dodd J. M. Newman A. Moran L. J. Deussen A. R. Grivell R. M. Yelland L. N. (2016). The effect of antenatal dietary and lifestyle advice for women who are overweight or obese on emotional well-being: the LIMIT randomized trial. *Acta Obstet. Gynecol. Scand.* 95 309–318. 10.1111/aogs.12832 26618547

[B29] Dunn G. A. Bale T. L. (2009). Maternal high-fat diet promotes body length increases and insulin insensitivity in second-generation mice. *Endocrinology* 150 4999–5009. 10.1210/en.2009-0500 19819967 PMC2775990

[B30] Dunn G. A. Bale T. L. (2011). Maternal high-fat diet effects on third-generation female body size via the paternal lineage. *Endocrinology* 152 2228–2236. 10.1210/en.2010-1461 21447631 PMC3100614

[B31] Dunn G. A. Morgan C. P. Bale T. L. (2011). Sex-specificity in transgenerational epigenetic programming. *Horm. Behav.* 59 290–295. 10.1016/j.yhbeh.2010.05.004 20483359

[B32] Elahi M. M. Cagampang F. R. Mukhtar D. Anthony F. W. Ohri S. K. Hanson M. A. (2009). Long-term maternal high-fat feeding from weaning through pregnancy and lactation predisposes offspring to hypertension, raised plasma lipids and fatty liver in mice. *Br. J. Nutr.* 102 514–519. 10.1017/s000711450820749x 19203419

[B33] Fernandez-Twinn D. S. Gascoin G. Musial B. Carr S. Duque-Guimaraes D. Blackmore H. L. (2017). Exercise rescues obese mothers’ insulin sensitivity, placental hypoxia and male offspring insulin sensitivity. *Sci. Rep.* 7:44650. 10.1038/srep44650 PMC5349590 28291256

[B34] Freedman D. S. Mei Z. Srinivasan S. R. Berenson G. S. Dietz W. H. (2007). Cardiovascular risk factors and excess adiposity among overweight children and adolescents: the bogalusa heart study. *J. Pediatr.* 150 12–17.e2. 17188605 10.1016/j.jpeds.2006.08.042

[B35] Gage S. H. Lawlor D. A. Tilling K. Fraser A. (2013). Associations of maternal weight gain in pregnancy with offspring cognition in childhood and adolescence: findings from the Avon Longitudinal Study of Parents and Children. *Am. J. Epidemiol.* 177 402–410. 10.1093/aje/kws239 23388581 PMC3581073

[B36] Gerstein H. Hullinger R. Lindstrom M. J. Burger C. (2013). A behavioral paradigm to evaluate hippocampal performance in aged rodents for pharmacological and genetic target validation. *PLoS One* 8:e62360. 10.1371/journal.pone.0062360 23667471 PMC3646843

[B37] Ghosh P. Bitsanis D. Ghebremeskel K. Crawford M. A. Poston L. (2001). Abnormal aortic fatty acid composition and small artery function in offspring of rats fed a high fat diet in pregnancy. *J. Physiol.* 533 815–822. 10.1111/j.1469-7793.2001.00815.x 11410637 PMC2278671

[B38] Godfrey K. M. Reynolds R. M. Prescott S. L. Nyirenda M. Jaddoe V. W. Eriksson J. G. (2017). Influence of maternal obesity on the long-term health of offspring. *Lancet Diabetes Endocrinol.* 5 53–64. 27743978 10.1016/S2213-8587(16)30107-3 PMC5245733

[B39] Graf A. E. Lallier S. W. Waidyaratne G. Thompson M. D. Tipple T. E. Hester M. E. (2016). Maternal high fat diet exposure is associated with increased hepcidin levels, decreased myelination, and neurobehavioral changes in male offspring. *Brain Behav. Immun.* 58 369–378. 10.1016/j.bbi.2016.08.005 27519153 PMC5611850

[B40] Grant W. B. (1999). Dietary links to Alzheimer’s disease: 1999 update. *J. Alzheimers Dis. JAD* 1 197–201. 10.3233/jad-1999-14-501 12214118

[B41] Gregersen S. Dyrskog S. E. Storlien L. H. Hermansen K. (2005). Comparison of a high saturated fat diet with a high carbohydrate diet during pregnancy and lactation: effects on insulin sensitivity in offspring of rats. *Metabolism* 54 1316–1322. 10.1016/j.metabol.2005.04.020 16154430

[B42] Gregor L. Remington P. L. Lindberg S. Ehrenthal D. (2016). Prevalence of Pre-pregnancy Obesity, 2011-2014. *WMJ* 115 228–232. 29095583 PMC5298836

[B43] Gregorio B. M. Souza-Mello V. Carvalho J. J. Mandarim-de-Lacerda C. A. Aguila M. B. (2010). Maternal high-fat intake predisposes nonalcoholic fatty liver disease in C57BL/6 offspring. *Am. J. Obstetr. Gynecol.* 203 e1–e8. 10.1016/j.ajog.2010.06.042 20822767

[B44] Guillemot-Legris O. Muccioli G. G. (2017). Obesity-Induced neuroinflammation: beyond the hypothalamus. *Trends Neurosci.* 40 237–253. 10.1016/j.tins.2017.02.005 28318543

[B45] Gustafson D. R. (2012). Adiposity and cognitive decline: underlying mechanisms. *J. Alzheimers Dis. JAD* 30(Suppl. 2) S97–S112. 22543853 10.3233/JAD-2012-120487

[B46] Han S. Middleton P. Crowther C. A. (2012). Exercise for pregnant women for preventing gestational diabetes mellitus. *Cochrane Database Syst. Rev.* 7:Cd009021. 10.1002/14651858.CD009021.pub2 PMC11534277 22786521

[B47] Hardy J. A. Higgins G. A. J. S. (1992). Alzheimer’s disease: the amyloid cascade hypothesis. *Science* 256 184–186. 1566067 10.1126/science.1566067

[B48] Hawkes C. A. Gentleman S. M. Nicoll J. A. Carare R. O. (2015). Prenatal high-fat diet alters the cerebrovasculature and clearance of β-amyloid in adult offspring. *J. Pathol.* 235 619–631. 10.1002/path.4468 25345857

[B49] Heslehurst N. Rankin J. Wilkinson J. R. Summerbell C. D. (2010). A nationally representative study of maternal obesity in England, UK: trends in incidence and demographic inequalities in 619 323 births, 1989-2007. *Int. J. Obes. (2005)* 34 420–428. 10.1038/ijo.2009.250 20029373

[B50] Hinkle S. N. Schieve L. A. Stein A. D. Swan D. W. Ramakrishnan U. Sharma A. J. (2012). Associations between maternal prepregnancy body mass index and child neurodevelopment at 2 years of age. *Int. J. Obes. (2005)* 36 1312–1319. 10.1038/ijo.2012.143 22964791 PMC4583192

[B51] Hooijmans C. R. Kiliaan A. J. (2008). Fatty acids, lipid metabolism and Alzheimer pathology. *Eur. J. Pharmacol.* 585 176–196. 10.1016/j.ejphar.2007.11.081 18378224

[B52] Howie G. J. Sloboda D. M. Kamal T. Vickers M. H. (2009). Maternal nutritional history predicts obesity in adult offspring independent of postnatal diet. *J. Physiol.* 587 905–915. 10.1113/jphysiol.2008.163477 19103681 PMC2669979

[B53] Hruby A. Hu F. B. (2015). The epidemiology of obesity: a big picture. *Pharmacoeconomics* 33 673–689. 10.1007/s40273-014-0243-x 25471927 PMC4859313

[B54] Huang L. Yu X. Keim S. Li L. Zhang L. Zhang J. (2014). Maternal prepregnancy obesity and child neurodevelopment in the Collaborative Perinatal Project. *Int. J. Epidemiol.* 43 783–792. 10.1093/ije/dyu030 24569381

[B55] Huda S. S. Brodie L. E. Sattar N. (2010). Obesity in pregnancy: prevalence and metabolic consequences. *Semin. Fetal Neonatal Med.* 15 70–76. 10.1016/j.siny.2009.09.006 19896913

[B56] Ishii T. (1966). Distribution of Alzheimer’s neurofibrillary changes in the brain stem and hypothalamus of senile dementia. *Acta Neuropathol.* 6 181–187. 10.1007/bf00686763 5963288

[B57] Janthakhin Y. Rincel M. Costa A. M. Darnaudéry M. Ferreira G. (2017). Maternal high-fat diet leads to hippocampal and amygdala dendritic remodeling in adult male offspring. *Psychoneuroendocrinology* 83 49–57. 10.1016/j.psyneuen.2017.05.003 28595087

[B58] Jefferson A. L. Massaro J. M. Wolf P. A. Seshadri S. Au R. Vasan R. S. (2007). Inflammatory biomarkers are associated with total brain volume: the Framingham Heart Study. *Neurology* 68 1032–1038. 10.1212/01.wnl.0000257815.20548.df 17389308 PMC2758770

[B59] Jenabi E. Bashirian S. Khazaei S. Basiri Z. (2019). The maternal prepregnancy body mass index and the risk of attention deficit hyperactivity disorder among children and adolescents: a systematic review and meta-analysis. *Korean J. Pediatr.* 62 374–379. 10.3345/kjp.2019.00185 31208166 PMC6801198

[B60] Kang S. S. Kurti A. Fair D. A. Fryer J. D. (2014). Dietary intervention rescues maternal obesity induced behavior deficits and neuroinflammation in offspring. *J. Neuroinflammation* 11:156. 10.1186/s12974-014-0156-9 PMC4172780 25212412

[B61] Keim S. A. Pruitt N. T. (2012). Gestational weight gain and child cognitive development. *Int. J. Epidemiol.* 41 414–422. 10.1093/ije/dyr229 22314966

[B62] Khan I. Y. Dekou V. Douglas G. Jensen R. Hanson M. A. Poston L. (2005). A high-fat diet during rat pregnancy or suckling induces cardiovascular dysfunction in adult offspring. *Am. J. Physiol. Regul. Integr. Comp. Physiol.* 288 R127–R133. 15308487 10.1152/ajpregu.00354.2004

[B63] Killin L. O. Starr J. M. I Shiue J. Russ T. C. (2016). Environmental risk factors for dementia: a systematic review. *BMC Geriatr.* 16:175. 10.1186/s12877-016-0342-y 27729011 PMC5059894

[B64] Kim J. Basak J. M. Holtzman D. M. (2009a). The role of apolipoprotein E in Alzheimer’s disease. *Neuron* 63 287–303. 19679070 10.1016/j.neuron.2009.06.026 PMC3044446

[B65] Kim J. Castellano J. M. Jiang H. Basak J. M. Parsadanian M. Pham V. (2009b). Overexpression of low-density lipoprotein receptor in the brain markedly inhibits amyloid deposition and increases extracellular A beta clearance. *Neuron* 64 632–644. 10.1016/j.neuron.2009.11.013 20005821 PMC2787195

[B66] Kim T. W. Park H. S. (2018). Physical exercise improves cognitive function by enhancing hippocampal neurogenesis and inhibiting apoptosis in male offspring born to obese mother. *Behav. Brain Res.* 347 360–367. 10.1016/j.bbr.2018.03.018 29551732

[B67] Kominiarek M. A. Chauhan S. P. (2016). Obesity before, during, and after pregnancy: a review and comparison of five national guidelines. *Am. J. Perinatol.* 33 433–441. 10.1055/s-0035-1567856 26588260

[B68] Kominiarek M. A. Peaceman A. M. (2017). Gestational weight gain. *Am. J. Obstetr. Gynecol.* 217 642–651. 10.1016/j.ajog.2017.05.040 PMC5701873 28549978

[B69] Kong L. Chen X. Gissler M. Lavebratt C. (2020). Relationship of prenatal maternal obesity and diabetes to offspring neurodevelopmental and psychiatric disorders: a narrative review. *Int. J. Obes.* 44 1981–2000. 10.1038/s41366-020-0609-4 32494038 PMC7508672

[B70] Kral T. V. Rauh E. M. (2010). Eating behaviors of children in the context of their family environment. *Physiol. Behav.* 100 567–573. 10.1016/j.physbeh.2010.04.031 20457172 PMC2896260

[B71] Kullmann S. Callaghan M. F. Heni M. Weiskopf N. Scheffler K. Haring H. U. (2016). Specific white matter tissue microstructure changes associated with obesity. *NeuroImage* 125 36–44. 10.1016/j.neuroimage.2015.10.006 26458514 PMC4692452

[B72] Kuo H. K. Yen C. J. Chang C. H. Kuo C. K. Chen J. H. Sorond F. (2005). Relation of C-reactive protein to stroke, cognitive disorders, and depression in the general population: systematic review and meta-analysis. *Lancet Neurol.* 4 371–380. 10.1016/s1474-4422(05)70099-5 15907742

[B73] Lahiri D. K. Zawia N. H. Greig N. H. Sambamurti K. Maloney B. (2008). Early-life events may trigger biochemical pathways for Alzheimer’s disease: the “LEARn” model. *Biogerontology* 9 375–379. 10.1007/s10522-008-9162-6 18668339 PMC4841617

[B74] Laitinen M. H. Ngandu T. Rovio S. Helkala E. L. Uusitalo U. Viitanen M. (2006). Fat intake at midlife and risk of dementia and Alzheimer’s disease: a population-based study. *Dement. Geriatr. Cogn. Disord.* 22 99–107. 10.1159/000093478 16710090

[B75] Lane-Donovan C. E. Philips G. T. Herz J. (2014). More than cholesterol transporters: lipoprotein receptors in CNS function and neurodegeneration. *Neuron* 83 771–787. 10.1016/j.neuron.2014.08.005 25144875 PMC4240629

[B76] Lawlor D. A. Smith G. D. O’Callaghan M. Alati R. Mamun A. A. Williams G. M. (2007). Epidemiologic evidence for the fetal overnutrition hypothesis: findings from the mater-university study of pregnancy and its outcomes. *Am. J. Epidemiol.* 165 418–424. 10.1093/aje/kwk030 17158475

[B77] Lépinay A. L. Larrieu T. Joffre C. Acar N. Gárate I. Castanon N. (2015). Perinatal high-fat diet increases hippocampal vulnerability to the adverse effects of subsequent high-fat feeding. *Psychoneuroendocrinology* 53 82–93. 10.1016/j.psyneuen.2014.12.008 25614359

[B78] Lewis A. R. Singh S. Youssef F. F. (2019). Cafeteria-diet induced obesity results in impaired cognitive functioning in a rodent model. *Heliyon* 5:e01412. 10.1016/j.heliyon.2019.e01412 30976688 PMC6441847

[B79] Li L. Lagerberg T. Chang Z. Cortese S. Rosenqvist M. A. Almqvist C. (2020). Maternal pre-pregnancy overweight/obesity and the risk of attention-deficit/hyperactivity disorder in offspring: a systematic review, meta-analysis and quasi-experimental family-based study. *Int. J. Epidemiol.* 49 857–875. 10.1093/ije/dyaa040 32337582 PMC7394963

[B80] Li M. Fallin M. D. Riley A. Landa R. Walker S. O. Silverstein M. (2016). The association of maternal obesity and diabetes with autism and other developmental disabilities. *Pediatrics* 137:e20152206. 10.1542/peds.2015-2206 26826214 PMC4732357

[B81] Li Y. Dai Q. Jackson J. C. Zhang J. (2008). Overweight is associated with decreased cognitive functioning among school-age children and adolescents. *Obesity (Silver Spring, Md.)* 16 1809–1815. 10.1038/oby.2008.296 18551126

[B82] Liang C. Oest M. E. Prater M. R. (2009). Intrauterine exposure to high saturated fat diet elevates risk of adult-onset chronic diseases in C57BL/6 mice. *Birth Defects Res. B Dev. Reprod. Toxicol.* 86 377–384. 10.1002/bdrb.20206 19750488

[B83] Lindberg S. Anderson C. Pillai P. Tandias A. Arndt B. Hanrahan L. (2016). Prevalence and predictors of unhealthy weight gain in pregnancy. *WMJ* 115 233–237. 29095584 PMC5313046

[B84] Liu Q. Trotter J. Zhang J. Peters M. M. Cheng H. Bao J. (2010). Neuronal LRP1 knockout in adult mice leads to impaired brain lipid metabolism and progressive, age-dependent synapse loss and neurodegeneration. *J. Neurosci.* 30 17068–17078. 10.1523/jneurosci.4067-10.2010 21159977 PMC3146802

[B85] Liu X. Li X. Xia B. Jin X. Zeng Z. Yan S. (2020). High-Fiber diet restores maternal obesity-induced cognitive and social behavioral deficits in offspring via regulating gut microbiota-metabolites-brain axis. *bioRxiv* [preprint]. 10.1101/2020.07.16.206714

[B86] Lloret A. Monllor P. Esteve D. Cervera-Ferri A. Lloret A. (2019). Obesity as a risk factor for Alzheimer’s disease: implication of leptin and glutamate. 13:508 10.3389/fnins.2019.00508 PMC6540965 31191220

[B87] Loche E. Blackmore H. L. Carpenter A. A. Beeson J. H. Pinnock A. Ashmore T. J. (2018). Maternal diet-induced obesity programmes cardiac dysfunction in male mice independently of post-weaning diet. *Cardiovasc. Res.* 114 1372–1384. 10.1093/cvr/cvy082 29635288 PMC6054211

[B88] Luchsinger J. A. Tang M. X. Shea S. Mayeux R. (2002). Caloric intake and the risk of Alzheimer disease. *Arch. Neurol.* 59 1258–1263. 10.1001/archneur.59.8.1258 12164721

[B89] Mahley R. W. (2016a). Apolipoprotein E: from cardiovascular disease to neurodegenerative disorders. *J. Mol. Med. (Berlin, Germany)* 94 739–746. 10.1007/s00109-016-1427-y 27277824 PMC4921111

[B90] Mahley R. W. (2016b). Central nervous system lipoproteins: ApoE and regulation of cholesterol metabolism. *Arterioscler. Thromb. Vasc. Biol.* 36 1305–1315. 10.1161/atvbaha.116.307023 27174096 PMC4942259

[B91] Martin S. A. Jameson C. H. Allan S. M. Lawrence C. B. (2014). Maternal high-fat diet worsens memory deficits in the triple-transgenic (3xTgAD) mouse model of Alzheimer’s disease. *PLoS One* 9:e99226. 10.1371/journal.pone.0099226 24918775 PMC4053375

[B92] McAninch D. Bianco-Miotto T. Gatford K. L. Leemaqz S. Y. Andraweera P. H. Garrett A. (2020). The metabolic syndrome in pregnancy and its association with child telomere length. *Diabetologia* 63 2140–2149. 10.1007/s00125-020-05242-0 32728890

[B93] McMillen I. C. Adam C. L. Muhlhausler B. S. (2005). Early origins of obesity: programming the appetite regulatory system. *J. Physiol.* 565 9–17. 10.1113/jphysiol.2004.081992 15705647 PMC1464497

[B94] Miller A. A. Spencer S. J. (2014). Obesity and neuroinflammation: a pathway to cognitive impairment. *Brain Behav. Immun.* 42 10–21. 10.1016/j.bbi.2014.04.001 24727365

[B95] Miller D. B. O’Callaghan J. P. (2008). Do early-life insults contribute to the late-life development of Parkinson and Alzheimer diseases? *Metabolism* 57(Suppl. 2) S44–S49. 18803966 10.1016/j.metabol.2008.07.011

[B96] Moreton E. Baron P. Tiplady S. McCall S. Clifford B. Langley-Evans S. C. (2019). Impact of early exposure to a cafeteria diet on prefrontal cortex monoamines and novel object recognition in adolescent rats. *Behav. Brain Res.* 363 191–198. 10.1016/j.bbr.2019.02.003 30735761

[B97] Morris M. J. Chen H. (2009). Established maternal obesity in the rat reprograms hypothalamic appetite regulators and leptin signaling at birth. *Int. J. Obes. (2005)* 33 115–122. 10.1038/ijo.2008.213 18982008

[B98] Naef L. Moquin L. Dal Bo G. Giros B. Gratton A. Walker C. D. (2011). Maternal high-fat intake alters presynaptic regulation of dopamine in the nucleus accumbens and increases motivation for fat rewards in the offspring. *Neuroscience* 176 225–236. 10.1016/j.neuroscience.2010.12.037 21187125

[B99] Naef L. Srivastava L. Gratton A. Hendrickson H. Owens S. M. Walker C. D. (2008). Maternal high fat diet during the perinatal period alters mesocorticolimbic dopamine in the adult rat offspring: reduction in the behavioral responses to repeated amphetamine administration. *Psychopharmacology* 197 83–94. 10.1007/s00213-007-1008-4 18004547

[B100] Nathanielsz P. W. Ford S. P. Long N. M. Vega C. C. Reyes-Castro L. A. Zambrano E. (2013). Interventions to prevent adverse fetal programming due to maternal obesity during pregnancy. *Nutr. Rev.* 71(Suppl. 1) S78–S87. 24147928 10.1111/nure.12062 PMC4193906

[B101] Neggers Y. H. Goldenberg R. L. Ramey S. L. Cliver S. P. (2003). Maternal prepregnancy body mass index and psychomotor development in children. *Acta Obstet. Gynecol. Scand.* 82 235–240. 10.1034/j.1600-0412.2003.00090.x 12694119

[B102] Ng M. Fleming T. Robinson M. Thomson B. Graetz N. Margono C. (2014). Global, regional, and national prevalence of overweight and obesity in children and adults during 1980-2013: a systematic analysis for the Global Burden of Disease Study 2013. *Lancet (London, England)* 384 766–781. 10.1016/S0140-6736(14)60460-8 PMC4624264 24880830

[B103] Niculescu M. D. Lupu D. S. (2009). High fat diet-induced maternal obesity alters fetal hippocampal development. *Int. J. Dev. Neurosci.* 27 627–633. 10.1016/j.ijdevneu.2009.08.005 19695321 PMC2754591

[B104] Nizari S. Carare R. O. Hawkes C. A. (2016). Increased Aβ pathology in aged Tg2576 mice born to mothers fed a high fat diet. *Sci. Rep.* 6:21981. 10.1038/srep21981 PMC4766411 26911528

[B105] Ogden C. L. Carroll M. D. Kit B. K. Flegal K. M. (2014). Prevalence of childhood and adult obesity in the United States, 2011-2012. *JAMA* 311 806–814. 10.1001/jama.2014.732 24570244 PMC4770258

[B106] Oken E. Taveras E. M. Kleinman K. P. Rich-Edwards J. W. Gillman M. W. (2007). Gestational weight gain and child adiposity at age 3 years. *Am. J. Obstet. Gynecol.* 196 e1–e8. 10.1016/j.ajog.2006.11.027 PMC1899090 17403405

[B107] Page K. C. Jones E. K. Anday E. K. (2014). Maternal and postweaning high-fat diets disturb hippocampal gene expression, learning, and memory function. *Am. J. Physiol. Regul. Integr. Comp. Physiol.* 306 R527–R537. 24523341 10.1152/ajpregu.00319.2013

[B108] Panza F. Solfrizzi V. Colacicco A. D’introno A. Capurso C. Torres F. (2004). Mediterranean diet and cognitive decline. *Public Health Nutr.* 7 959–963. 15482625 10.1079/phn2004561

[B109] Peleg-Raibstein D. Luca E. Wolfrum C. (2012). Maternal high-fat diet in mice programs emotional behavior in adulthood. *Behav. Brain Res.* 233 398–404. 10.1016/j.bbr.2012.05.027 22633920

[B110] Peleg-Raibstein D. Sarker G. Litwan K. Kramer S. D. Ametamey S. M. Schibli R. (2016). Enhanced sensitivity to drugs of abuse and palatable foods following maternal overnutrition. *Transl. Psychiatry* 6:e911. 10.1038/tp.2016.176 27701408 PMC5315546

[B111] Poston L. Bell R. Briley A. L. Godfrey K. M. Nelson S. M. Oteng-Ntim E. (2017). “Improving pregnancy outcome in obese women: the UK Pregnancies Better Eating and Activity randomised controlled Trial,” in *Programme Grants for Applied Research, No. 5.10*, (Southampton, UK: NIHR Journals Library). 28671801

[B112] Poston L. Bell R. Croker H. Flynn A. C. Godfrey K. M. Goff L. (2015). Effect of a behavioural intervention in obese pregnant women (the UPBEAT study): a multicentre, randomised controlled trial. *Lancet Diabetes Endocrinol.* 3 767–777. 10.1016/s2213-8587(15)00227-2 26165396

[B113] Pugh S. J. Richardson G. A. Hutcheon J. A. Himes K. P. Brooks M. M. Day N. L. (2015). Maternal obesity and excessive gestational weight gain are associated with components of child cognition. *J. Nutr.* 145 2562–2569. 10.3945/jn.115.215525 26423736 PMC4620725

[B114] Pulgarón E. R. (2013). Childhood obesity: a review of increased risk for physical and psychological comorbidities. *Clin. Ther.* 35 A18–A32. 23328273 10.1016/j.clinthera.2012.12.014 PMC3645868

[B115] Reynolds R. M. Allan K. M. Raja E. A. Bhattacharya S. McNeill G. Hannaford P. C. (2013). Maternal obesity during pregnancy and premature mortality from cardiovascular event in adult offspring: follow-up of 1 323 275 person years. *BMJ Clin. Res. ed.* 347:f4539. 10.1136/bmj.f4539 23943697 PMC3805484

[B116] Ribeiro A. Batista T. H. Veronesi V. B. Giusti-Paiva A. Vilela F. C. (2018). Cafeteria diet during the gestation period programs developmental and behavioral courses in the offspring. *Int. J. Dev. Neurosci.* 68 45–52. 10.1016/j.ijdevneu.2018.05.001 29730049

[B117] Robb J. L. Messa I. Lui E. Yeung D. Thacker J. Satvat E. (2017). A maternal diet high in saturated fat impairs offspring hippocampal function in a sex-specific manner. *Behav. Brain Res.* 326 187–199. 10.1016/j.bbr.2017.02.049 28259676

[B118] Robinson S. L. Ghassabian A. Sundaram R. Trinh M.-H. Lin T.-C. Bell E. M. (2020). Parental weight status and offspring behavioral problems and psychiatric symptoms. *J. Pediatr.* 220 227–236.e1. 32067780 10.1016/j.jpeds.2020.01.016 PMC7186145

[B119] Rodriguez A. (2010). Maternal pre-pregnancy obesity and risk for inattention and negative emotionality in children. *J. Child Psychol. Psychiatry* 51 134–143. 10.1111/j.1469-7610.2009.02133.x 19674195

[B120] Rodriguez A. Miettunen J. Henriksen T. B. Olsen J. Obel C. Taanila A. (2008). Maternal adiposity prior to pregnancy is associated with ADHD symptoms in offspring: evidence from three prospective pregnancy cohorts. *Int. J. Obes. (2005)* 32 550–557. 10.1038/sj.ijo.0803741 17938639

[B121] Samuelsson A. M. Matthews P. A. Argenton M. Christie M. R. McConnell J. M. Jansen E. H. (2008). Diet-induced obesity in female mice leads to offspring hyperphagia, adiposity, hypertension, and insulin resistance: a novel murine model of developmental programming. *Hypertension* 51 383–392. 10.1161/hypertensionaha.107.101477 18086952

[B122] Saper C. B. German D. C. (1987). Hypothalamic pathology in Alzheimer’s disease. *Neurosci. Lett.* 74 364–370. 2436113 10.1016/0304-3940(87)90325-9

[B123] Sarker G. Berrens R. von Arx J. Pelczar P. Reik W. Wolfrum C. (2018). Transgenerational transmission of hedonic behaviors and metabolic phenotypes induced by maternal overnutrition. *Transl. Psychiatry* 8:195. 10.1038/s41398-018-0243-2 PMC6185972 30315171

[B124] Sarker G. Litwan K. Kastli R. Peleg-Raibstein D. (2019a). Maternal overnutrition during critical developmental periods leads to different health adversities in the offspring: relevance of obesity, addiction and schizophrenia. *Sci. Rep.* 9:17322. 10.1038/s41598-019-53652-x PMC6872534 31754139

[B125] Sarker G. Peleg-Raibstein D. (2018). Maternal overnutrition induces long-term cognitive deficits across several generations. *Nutrients* 11:7. 10.3390/nu11010007 30577472 PMC6356622

[B126] Sarker G. Sun W. Rosenkranz D. Pelczar P. Opitz L. Efthymiou V. (2019b). Maternal overnutrition programs hedonic and metabolic phenotypes across generations through sperm tsRNAs. *Proc. Natl. Acad. Sci. U.S.A.* 116 10547–10556. 10.1073/pnas.1820810116 31061112 PMC6534971

[B127] Schain M. Kreisl W. C. (2017). Neuroinflammation in neurodegenerative disorders-a review. *Curr. Neurol. Neurosc. Rep.* 17:25. 10.1007/s11910-017-0733-2 28283959

[B128] Sellbom K. S. Gunstad J. (2012). Cognitive function and decline in obesity. *J. Alzheimers Dis. JAD* 30(Suppl. 2) S89–S95. 22258511 10.3233/JAD-2011-111073

[B129] Sen S. Simmons R. A. (2010). Maternal antioxidant supplementation prevents adiposity in the offspring of Western diet-fed rats. *Diabetes* 59 3058–3065. 10.2337/db10-0301 20823102 PMC2992766

[B130] Sheikh A. B. Nasrullah A. Haq S. Akhtar A. Ghazanfar H. Nasir A. (2017). The interplay of genetics and environmental factors in the development of obesity. *Cureus* 9:e1435. 10.7759/cureus.1435 PMC5587406 28924523

[B131] Siemelink M. Verhoef A. Dormans J. A. Span P. N. Piersma A. H. (2002). Dietary fatty acid composition during pregnancy and lactation in the rat programs growth and glucose metabolism in the offspring. *Diabetologia* 45 1397–1403. 10.1007/s00125-002-0918-2 12378380

[B132] Simar D. Chen H. Lambert K. Mercier J. Morris M. J. (2011). Interaction between maternal obesity and post-natal over-nutrition on skeletal muscle metabolism. *Nutr. Metab. Cardiovasc. Dis.* 3:3. 10.1016/j.numecd.2010.11.007 21208789

[B133] Stacey D. Ciobanu L. G. Baune B. T. (2015). A systematic review on the association between inflammatory genes and cognitive decline in non-demented elderly individuals. *Eur. Neuropsychopharmacol.* 27 568–588. 10.1016/j.euroneuro.2015.12.017 26718789

[B134] Strakovsky R. S. Zhang X. Zhou D. Pan Y. X. (2011). Gestational high fat diet programs hepatic phosphoenolpyruvate carboxykinase gene expression and histone modification in neonatal offspring rats. *J. Physiol.* 589 2707–2717. 10.1113/jphysiol.2010.203950 21486814 PMC3112549

[B135] Stranahan A. M. Mattson M. P. (2008). Impact of energy intake and expenditure on neuronal plasticity. *Neuromolecular Med.* 10 209–218. 10.1007/s12017-008-8043-0 18543119 PMC2635925

[B136] Stranahan A. M. Norman E. D. Lee K. Cutler R. G. Telljohann R. S. Egan J. M. (2008). Diet-induced insulin resistance impairs hippocampal synaptic plasticity and cognition in middle-aged rats. *Hippocampus* 18 1085–1088. 10.1002/hipo.20470 18651634 PMC2694409

[B137] Tamashiro K. L. Terrillion C. E. Hyun J. Koenig J. I. Moran T. H. (2009). Prenatal stress or high-fat diet increases susceptibility to diet-induced obesity in rat offspring. *Diabetes* 58 1116–1125. 10.2337/db08-1129 19188431 PMC2671057

[B138] Tanda R. Salsberry P. J. Reagan P. B. Fang M. Z. (2013). The impact of prepregnancy obesity on children’s cognitive test scores. *Matern. Child Health J.* 17 222–229. 10.1007/s10995-012-0964-4 22350633 PMC3370113

[B139] Thangaratinam S. (2015). Diet and lifestyle interventions for obese pregnant women. *Lancet Diabetes Endocrinol.* 3 748–749. 10.1016/s2213-8587(15)00253-3 26165397

[B140] Tolppanen A. M. Ahonen R. Koponen M. Lavikainen P. Purhonen M. Taipale H. (2016). Month and season of birth as a risk factor for alzheimer’s disease: a nationwide nested case-control study. *J. Prev. Med. Public Health* 49 134–138. 10.3961/jpmph.16.018 27055550 PMC4829371

[B141] Tozuka Y. Kumon M. Wada E. Onodera M. Mochizuki H. Wada K. (2010). Maternal obesity impairs hippocampal BDNF production and spatial learning performance in young mouse offspring. *Neurochem. Int.* 57 235–247. 10.1016/j.neuint.2010.05.015 20538025

[B142] Tozuka Y. Wada E. Wada K. (2009). Diet-induced obesity in female mice leads to peroxidized lipid accumulations and impairment of hippocampal neurogenesis during the early life of their offspring. *FASEB J.* 23 1920–1934. 10.1096/fj.08-124784 19158155

[B143] Van Lieshout R. J. (2013). Role of maternal adiposity prior to and during pregnancy in cognitive and psychiatric problems in offspring. *Nutr. Rev.* 71(Suppl. 1) S95–S101. 24147931 10.1111/nure.12059

[B144] Van Lieshout R. J. Taylor V. H. Boyle M. H. (2011). Pre-pregnancy and pregnancy obesity and neurodevelopmental outcomes in offspring: a systematic review. *Obes. Rev.* 12 e548–e559. 21414129 10.1111/j.1467-789X.2010.00850.x

[B145] Vega C. C. Reyes-Castro L. A. Bautista C. J. Larrea F. Nathanielsz P. W. Zambrano E. (2015). Exercise in obese female rats has beneficial effects on maternal and male and female offspring metabolism. *Int. J. Obes.* 39 712–719. 10.1038/ijo.2013.150 23949616 PMC3925765

[B146] Vickers M. H. Gluckman P. D. Coveny A. H. Hofman P. L. Cutfield W. S. Gertler A. (2005). Neonatal leptin treatment reverses developmental programming. *Endocrinology* 146 4211–4216. 10.1210/en.2005-0581 16020474

[B147] Vickers M. H. Sloboda D. M. (2012). Strategies for reversing the effects of metabolic disorders induced as a consequence of developmental programming. *Front. Physiol.* 3:242. 10.3389/fphys.2012.00242 22783205 PMC3387724

[B148] Vogt M. C. Paeger L. Hess S. Steculorum S. M. Awazawa M. Hampel B. (2014). Neonatal insulin action impairs hypothalamic neurocircuit formation in response to maternal high-fat feeding. *Cell* 156 495–509. 10.1016/j.cell.2014.01.008 24462248 PMC4101521

[B149] Vorhees C. V. Williams M. T. (2014). Assessing spatial learning and memory in rodents. *ILAR J.* 55 310–332. 10.1093/ilar/ilu013 25225309 PMC4240437

[B150] Vucetic Z. Kimmel J. Totoki K. Hollenbeck E. Reyes T. M. (2010). Maternal high-fat diet alters methylation and gene expression of dopamine and opioid-related genes. *Endocrinology* 151 4756–4764. 10.1210/en.2010-0505 20685869 PMC2946145

[B151] Wahlig J. L. Bales E. S. Jackman M. R. Johnson G. C. McManaman J. L. Maclean P. S. (2011). Impact of high-fat diet and obesity on energy balance and fuel utilization during the metabolic challenge of lactation. *Obesity (Silver Spring, Md.)* 30:196. 10.1038/oby.2011.196 PMC4109263 21720435

[B152] Whitaker R. C. (2004). Predicting preschooler obesity at birth: the role of maternal obesity in early pregnancy. *Pediatrics* 114 e29–e36. 15231970 10.1542/peds.114.1.e29

[B153] White C. L. Pistell P. J. Purpera M. N. Gupta S. Fernandez-Kim S. O. Hise T. L. (2009). Effects of high fat diet on Morris maze performance, oxidative stress, and inflammation in rats: contributions of maternal diet. *Neurobiol. Dis.* 35 3–13. 10.1016/j.nbd.2009.04.002 19374947 PMC2699188

[B154] WHO (2020). *Obesity and Overweight Fact Sheet.* Geneva: World Health Organization (WHO).

[B155] Wolfrum C. Peleg-Raibstein D. (2018). Maternal overnutrition leads to cognitive and neurochemical abnormalities in C57BL/6 mice. *Nutr. Neurosci.* 22 688–699. 10.1080/1028415x.2018.1432096 29390923

[B156] Wraw C. I Deary J. Der G. Gale C. R. (2018). Maternal and offspring intelligence in relation to BMI across childhood and adolescence. *Int. J. Obes.* 42 1610–1620. 10.1038/s41366-018-0009-1 29515207 PMC6002784

[B157] Wright T. M. King M. V. Davey W. G. Langley-Evans S. C. Voigt J. P. (2014). Impact of cafeteria feeding during lactation in the rat on novel object discrimination in the offspring. *Br. J. Nutr.* 112 1933–1937. 10.1017/s0007114514003134 25345572

[B158] Xian X. Liu T. Yu J. Wang Y. Miao Y. Zhang J. (2009). Presynaptic defects underlying impaired learning and memory function in lipoprotein lipase-deficient mice. *J. Neurosci.* 29 4681–4685. 10.1523/jneurosci.0297-09.2009 19357293 PMC6665748

[B159] Yamazaki Y. Zhao N. Caulfield T. R. Liu C.-C. Bu G. (2019). Apolipoprotein E and Alzheimer disease: pathobiology and targeting strategies. *Nat. Rev. Neurol.* 15 501–518. 10.1038/s41582-019-0228-7 31367008 PMC7055192

[B160] Zambrano E. Martínez-Samayoa P. M. Rodríguez-González G. L. Nathanielsz P. W. (2010). Dietary intervention prior to pregnancy reverses metabolic programming in male offspring of obese rats. *J. Physiol.* 588 1791–1799. 10.1113/jphysiol.2010.190033 20351043 PMC2887995

[B161] Zhang J. Liu Q. (2015). Cholesterol metabolism and homeostasis in the brain. *Protein Cell* 6 254–264. 10.1007/s13238-014-0131-3 25682154 PMC4383754

[B162] Zhang Z. Y. Zeng J. J. Kjaergaard M. Guan N. Raun K. Nilsson C. (2011). Effects of a maternal diet supplemented with chocolate and fructose beverage during gestation and lactation on rat dams and their offspring. *Clin. Exp. Pharmacol. Physiol.* 4 1440–1681. 10.1111/j.1440-1681.2011.05568.x 21722163

[B163] Zieba J. Uddin G. M. Youngson N. A. Karl T. Morris M. J. (2019). Long-term behavioural effects of maternal obesity in C57BL/6J mice. *Physiol. Behav.* 199 306–313. 10.1016/j.physbeh.2018.11.004 30414884

